# High Prevalence of Hookworm Species and Associated Factors among Soil-Transmitted Helminth-Infected Household Contacts in Burie Zuria District, Northwest Ethiopia: A Community-Based Cross-Sectional Study

**DOI:** 10.1155/2023/6553913

**Published:** 2023-01-07

**Authors:** Gedefaye Assefa, Megbaru Alemu, Animen Ayehu

**Affiliations:** ^1^Burie Primary Hospital, Amhara National Regional Health Bureau, Ethiopia; ^2^Department of Medical Laboratory Science, School of Health Sciences, College of Medicine and Health Sciences, Bahir Dar University, Bahir Dar, Ethiopia

## Abstract

**Background:**

Soil-transmitted helminths (STHs) are parasitic worms that are widely distributed in the tropical and subtropical regions. In Ethiopia, one of the tropical countries, STH infections are high and cause a huge burden. Several STH species show a pattern of household clustering with regard to prevalence and intensity. However, there is a scarcity of data on the status of STH infection among family contacts of STH-infected individuals in Ethiopia in general and in the study area in particular.

**Objective:**

This study is aimed at assessing the prevalence of soil-transmitted helminths and associated factors among STH-infected household contacts in Burie Zuria district, northwest Ethiopia.

**Method:**

A community-based cross-sectional study was conducted among 422 study participants from March to May 2021. Study participants were traced to their residences by following STH-infected patients. A convenient sampling technique was used to recruit the study participants. The stool samples were processed using duplicate Kato-Katz thick smears and a modified Ritchie's technique. The data were entered and analyzed using the Statistical Package for Social Sciences, version 26 of the software package. A *P* value <0.05 was considered a statistically significant association.

**Results:**

The overall prevalence of STHs was 36.5% (95% CI: 31.7%–41.5%). Two STHs, namely hookworm species (33.2%, 140/422) at (95% CI: 28.9%–37.8%) and *Ascaris lumbricoides* (4.3%, 18/422) at (95% CI: 2.7%–6.6%), were identified, with a double infection of 2.6% (4/154) at (95% CI: 1.0%–6.5%). Almost the majority (96.9%) of hookworm species and all *Ascaris lumbricoides* infections were categorized under a light intensity of infection. Family size >5 (AOR = 1.74; 95% CI: 1.15–2.60; *P* = 0.008) and lack of latrine facility (AOR = 1.86; 95% CI: 1.17–2.90; *P* = 0.02) were significantly associated with STH infections.

**Conclusion:**

A high prevalence of hookworms was found in the study area, where the majority of the study participants were adults. This finding may provide a basis for revising the school-based deworming programs that exclude the adult population. Public health interventions like accessing latrines, health education, and deworming programs should be regularly implemented for all age groups in the areas.

## 1. Background

Soil-transmitted helminths (STHs) are a group of parasitic worms that inhabit the human intestine and cause infection in humans. These include *Ascaris lumbricoides* (*A. lumbricoides*), *Trichuris trichiura* (*T. trichiura*), *Ancylostoma duodenale* (*A. duodenale*), and *Necator americanus* (*N. americanus*) [1]. They are widely distributed in tropical and subtropical regions, especially in Africa, Asia, and Latin America, which are associated with the poor socioeconomic status of developing countries [2] and with suitable environmental conditions that favor the transmission of these helminths [3, 4].

Soil-transmitted helminths affect more than 1.5 billion people worldwide, mostly in tropical regions in Africa, Asia, and Latin America [1]. *Ascaris lumbricoides* is the most prevalent STH, infecting about 1.2 billion people, followed by *T. trichiura* (infecting about 795 million people), and hookworms, which infect nearly 740 million people worldwide [5].

Sub-Saharan African countries, including Ethiopia, are the most endemic areas for STH infection, in which open defecation, walking barefoot, low latrine coverage, poor hygiene, and sanitation are common. Ethiopia, as part of sub-Saharan countries, has a high prevalence of STH, especially in the rural community [1].

Among many epidemiological determinants were as folows: socioeconomic, behavioral, environmental, service-related, and climatic factors are the main conceptual frames that can be associated with the transmission, burden, life cycle, and prevention of STH [6]. Climate is an important determinant for transmission of STH infections, with adequate moisture and a warm temperature being essential for the hatching or embryonation of STH eggs or for larval development in the soil. Transmission is common because of the free-living infective-stage development and the long-term survival of STHs. But very high and low land surface temperatures and extreme arid environments may limit STHs species transmission [7]. For example, the prevalence of *A. lumbricoides* and *T. trichiura* in Africa and the Middle East is generally less than 4% in areas with temperatures exceeding 35°C but the prevalence of hook worm infection is high at higher temperatures (up to 40°C) [8]. Poverty, inadequate water supplies, poor hygiene, and lack of sanitation are equally important determinants that are commonly coendemic for all STH species infections [7]. Household or family clustering of infection has also been reported for STH species [9]. However, household clustering may largely reflect transmission between individuals sharing a household; for example, *Ascaris* but not hookworms, infections were clustered within households in a study, which could be explained by hookworm species transmission occurring outside the household environment [10].

Ethiopia is the main endemic area for STHs infection in which open defecation, walking barefoot, low latrine coverage, poor hygiene, and poor sanitation are common. Epidemiological studies on STH so far in Ethiopia have focused on school-age children. Consequently, there is a scarcity of data on the status of household members of STH-infected patients. Community-level epidemiological data among household members of STH-infected patients will also be an important input for the targeted intervention.

The transmission pattern and epidemiology of STHs are also a potential areas of investigation in Ethiopia in general and in Burie Zuria district in particular. Moreover, the household clustering of STH infection is a research area that requires further efforts. Thus, the current study is aimed at assessing the prevalence of STH infections and associated risk factors among household contacts of STH confirmed-patients visiting selected health centers in Burie Zuria district, west Gojjam zone, northwest Ethiopia.

## 2. Materials and Methods

### 2.1. Study Area

The study was conducted in Burie Zuria district, located in west Gojjam zone, Amhara National Regional State, 411 kilometers northwest of Addis Ababa and 157 kilometers from Bahir Dar. The district has 22 rural kebele, 1 urban kebele, and 1 per capita urban kebele, and covers an area of 587.95 km^2^. It is also bordered on the south by the Abay River, on the west by Wemberma district, on the north by the Awi zone, and Sekela district, and on the east by Jabi Tehnan district. According to the Burie Zuria agricultural office report, the district receives a minimum annual rainfall of 1,200 mm per season, with a range of 900–1,400 mm. The altitude ranges from 700 to 2,300 meters above sea level, and 13.3% of the landmass is Kola, 66% is Woina Dega, and 20.7% is Dega. The total population of the district was 133,307, of which 67,025 were females and 66,282 were males (from the district health office). There are 5 health centers and 20 health posts.

### 2.2. Study Design and Period

A community-based cross-sectional study design was employed from March to May 2021.

### 2.3. Populations

#### 2.3.1. Source Population

All household members of patients requested for stool examination at health centers in the Burie Zuria district.

#### 2.3.2. Study Population

All household contacts of patients who were confirmed for STHs infection in Alefa and Tiatia health center catchments and who met eligibility criteria during the data collection period.

### 2.4. Inclusion and Exclusion Criteria

#### 2.4.1. Inclusion Criteria

All family members in the household during data collection time, ≥2 years old, volunteered to participate in the study, and were able to give stool samples were included.

#### 2.4.2. Exclusion Criteria

Household members of primary cases who have taken anti-helminthic drugs in the last two months, <2 years, live in another catchment, were critically ill and disabled (unable to respond to research questions were excluded.

### 2.5. Sample Size Determination and Sampling Technique

#### 2.5.1. Sample Size Determination

Since no previous studies are available about the prevalence of STH among household members of STHs infected individuals by contact tracing method, so we have taken 50% prevalence. Using a single population proportion, then the sample size was calculated by taking 95% CI, 5% margin of error with a 10% nonresponse rate;

Therefore, the total sample size was calculated as follows.

*n* = (*Z*_*α*/2_)^2^ × *P* (1 − *P*)/d^2^, where;

n = minimum sample size.

P = the proportion of contact tracing of family members (*P* = 0.5) which was taken as 50%, no previously known prevalence.

d = marginal error between the sample and population (0.05).

Z = critical value at 95% certainty (1.96) and considering 10% nonresponsive rate.

(1) $$\begin{array}{c} n=\frac{{\left(1.96\right)}^{2}\left(0.5\right){\left(1–0.5\right)}^{2}}{{\left(0.05\right)}^{2}}=384+\text{nonresponse rate }\left(10\%\right)=384+38.4=422. \end{array}$$

Therefore, 422 was the total sample size.

### 2.6. Sampling Technique

By using the convenience sampling technique, stool samples were collected from all confirmed STH-infected primary cases and family members until the maximum sample size was reached. Alefa and Tiatia health center catchments were selected by a simple random sampling technique from the district. The total population of the Alefa and Tiatia health center catchments was 23,292 and 20,770, respectively. The total number of households in both catchments was 9792. From this, the number of households in the Alefa catchment was 5,176 and 4616 in the Tiatia catchments. To accomplish a total of 422 study participants, 111 primary confirmed STH cases (index cases) were needed from 111 households (225 study participants from 57 households in Alefa and 197 study participants from 54 households in Tiatia) health center catchments, respectively, by the proportional allocation formula. A patient diagnosed positive for the STHs in both health centers was used to trace their family members for STH infection status. All household members of the primary case who fulfilled the inclusion criteria were included. Household contacts were accessed by documenting the primary cases' full addresses, like their kebele, got, full name, and phone number if they have one.

### 2.7. Data Collection

#### 2.7.1. Questionnaire

Data on sociodemographic characteristics, water, hygiene, and sanitary conditions were collected using a structured Amharic version of the questionnaire. The Amharic version of the questionnaire was administered by trained laboratory technicians through face-to-face interviews.

#### 2.7.2. Stool Sample Collection and Processing

The data collectors were consulted on how to collect a stool sample, provided clean and labeled stool cups, and instructed study participants to bring approximately 3 grams of their own stool. The stool samples were transported, using a cold box, to the Alefa and Tiatia health centers laboratories and examined within the same day. Most of the samples were collected in the morning because at this time the household members would be available. The samples were processed via a modified Ritchie's formol ether concentration technique and Kato-Katz (KK) methods for identification and intensity of STHs.

#### 2.7.3. Modified Ritchie's Formol Ether Concentration Technique

About 0.5 grams of stool sample was added to Ritchie'**s** tube, which contains 2.5 milliliters of formalin and 1 milliliter of ethyl acetate, and then it was mixed. The mixture was mixed gently and centrifuged at 1000 revolutions per minute for 3 minutes. After centrifugation, the supernatant was discarded and the sediment was mixed. Finally, one drop of the sediment was added to a slide for microscopic examination for STHs infection.

#### 2.7.4. Kato-Katz Method

In this method, a fresh stool sample was pressed through a mesh screen to remove large particles. About 41.7 mg of sieved stool was transferred to the template, which were put on a slide until the template hole was filled. Then, the template was removed from the microscope slide and the stool was covered with cellophane immersed in a glycerol-malachite green solution for 24 hours. The cellophane was pressed against another microscope slide to form a smear. Then, the pressing slide was removed sideways to prevent the detachment of the cellophane from the smear. The entire field of prepared slides was examined under the microscope within 45 to 60 minutes to prevent losing hookworm parasites. After examining the fields, the total number of eggs was counted and multiplied by a factor of 24 to obtain eggs per gram (EPG) of stool. The intensity of STHs was categorized as light (1–4999 EPG), moderate (5000–49999 EPG), and heavy (≥50,000) for *A. lumbricoides*); light (1–1999 EPG), moderate (2000–3999 EPG), and heavy (≥4000 EPG) for hookworms; light (1–999 EPG), moderate (1000–9999 EPG), and heavy (≥10,000) for *T. trichiura* [11].

### 2.8. Data Quality Control

The questionnaire and all necessary materials were checked before the actual data collection began. A detailed information was given to the data collectors on how to conduct the interview and how to collect the stool samples from the study participants. The quality of reagents was also checked and stored in a proper place. Sample collection and laboratory examination were performed on the same day to maintain the reliability of the study findings. The modified Riches techniques and Kato-Katz techniques were used to examine stools in the laboratory. In the Kato-Katz technique, double slides were counted for a single participant's sample, and an average egg count was taken to determine the intensity of the infection. Furthermore, 10% of the KK slides were chosen at random for quality control and reexamined by one laboratory personnel who checked blindly for previous results. Slides were rechecked for discordant content and managed.

### 2.9. Data Analysis

The data was entered and analyzed using the Statistical Package for Social Sciences (SPSS) version 26 software package. The prevalence of STHs was analyzed with descriptive statistics and reported in percent form, and the intensity of STHs was also analyzed using mean eggs per gram stool. Bivariate and multivariate logistic regressions were used to select the variables and determine the association between STHs infection and associated factors by computing odds ratios (ORs) at a 95% confidence level. In the bivariate analysis, variables with a *P* value of 0.25 were subjected to a multivariate logistic regression analysis model to identify predictor variables and control cofounders. The magnitude of the association was measured using the adjusted odds ratio (AOR) and 95% confidence interval (CI). A *P* value <0.05 was considered statistically significant.

## 3. Results

### 3.1. Sociodemographic Characteristics of Study Participants

A total of 422 study participants from 111 households in the Alefa and Tiatia health center catchment kebeles took part in the study. Even if there were 458 contacts of primary cases, 36 individuals were not included in the study due to several reasons, such as age less than 2 years, not volunteering to participate, absence during data collection, insufficient stool samples, and being unable to give a stool sample. Females constitute 54.3% (229/422) of the participants. The age of the study participants ranges from 2 to 80 years. The mean and median ages of participants were 27.1 and 22 years old, respectively. The majority of study participants (97.9%, 413/422) were from rural areas. Nearly half of the participants were farmers (51.2%, 216/422), followed by students (36.3%, 153/422). Around half (47.2%) of the participants were illiterate. Participants >15 years of age took the highest percentage, 61.1% (258/422), and those <5 years old took a lower percentage, 8.3% (35/422) (Table 1).

### 3.2. Prevalence of Intestinal Parasites

The overall prevalence of intestinal parasites was 47.2% (95% CI: 42.2%–51.9%). A total of six intestinal parasite species, namely: hookworm species (33.2%), *A. lumbricoides* (4.3%)*, E. histolytica/dispar* (4.5%), *G. intestinalis* (5.7%)*, Taenia* spp (0.9%), and *Hymenolepis nana* (*H. nana*) (0.5%), were identified in the study area. Hookworm species were the predominant parasites, with 33.2% (95% CI: 28.6%–37.7%). Double infection was reported in 4.0% (8/199) of study participants, with a 95% CI of 2.0%–7.4% for hookworm species and *A. lumbricoides* dominance (Table 2).

### 3.3. Prevalence of Soil-Transmitted Helminths

The overall prevalence of soil-transmitted helminths was 36.5% (154/422; 95% CI: 31.7%–41.5%). Hookworm species were the predominant STHs isolated, with a prevalence of 33.2% (140/422) with a 95% CI of 28.6%–37.7%, followed by *A. lumbricoides* (4.3%, 18/422) with a 95% CI of 2.6%–6.4%. From the total, the double infection of hookworm species and *A. lumbricoides* was found to be 2.6% (4/154) at a 95% CI of 1.0%–6.5% (Table 3).

The prevalence of STHs was higher among females (39.3%, 90/229) compared to males (33.2%, 64/194). Moreover, a higher prevalence of STH (45.3%) was recorded among household members with large family sizes (>5 members). Regarding residency, the majority of STH-infected participants were rural residents (35.8%, 151/413), and the majority of infected individuals were farmers (38.4%, 83/216). Distributions of STHs also vary across different factors. For example, a higher prevalence of STHs was observed among participants who did not have latrine around their house (47.9%, 46/96) and those who practiced open defecation (46%, 52/113). Moreover, STH prevalence was higher among participants who did not wash raw vegetables before eating (43.2%, 19/44) and those with poor handwashing habits before meal (52.4%, 11/21) (Table 4).

### 3.4. Factors Associated with STH Infections

Possible factors associated with soil-transmitted helminths were analyzed using bivariate and multivariate logistic regressions. Variables that were statistically associated with the prevalence of STHs and had a *P* value <0.25 in bivariate analysis were analyzed by multivariable analysis [12]. In line with it, in the bivariate logistic regression, family size greater than five, lack of latrine facility and open defecation showed significant association with STH infection. In the multivariate analysis, however, only lack of latrine facility (AOR: 1.86 (95% CI: 1.17-2.95) *P* = 0.008) and larger family size (AOR: 1.74 (95% CI: 1.15-2.63), *P* = 0.02) were significant predictors of STH infection (Table 5).

### 3.5. Intensity of Soil-Transmitted Helminth Infection

Regarding to infection intensity, of the total of 129 hookworm species infections detected in the Kato-Katz technique, 125 (96.9%) and 4 (3.1%) of the infected participants were categorized under light and moderate intensities, respectively, whereas all of the *A. lumbricoides* and mixed infections of study participants were grouped under light intensity. None of the infected study participants in all STHs infections were categorized under heavy infection intensity. The minimum and the maximum intensity of hookworm species infection was 74 and 3648 EPG, respectively. Whereas the average geometric mean was 927 EPG. Minimum and maximum intensity of infection with *A. lumbricoides* was 120 and 888 EPG, respectively. According to the STH infection intensity grading standards, the mean was 408 EPG (Table 6).

## 4. Discussion

Infection with soil-transmitted helminths in the rural community of the study area is still a major health problem and needs great concern. In the current study, the overall prevalence of STHs was 36.5% (95% CI: 31.7%–41.5%), which is in line with studies in Nigeria (34.2%) [13] and Kenya (39.3%) [14]. However, our finding was higher than the prevalence reported from Brazil (12.6%) [15], Malaysia (3.1%) [7], Thailand (18%) [16], and Bibugn Woreda, northwest Ethiopia (20.9%) [17]. This discrepancy in results may be due to the countries' better socioeconomic status than Ethiopia, and these countries may have strong prevention, control, and deworming programs for STH infections for their people. Another reason might be the fact that the study participants in our study had STH-infected contacts, and therefore they might share similar sources of infective stages of STHs. Contrarily, the current prevalence was lower than the findings from southwest Ethiopia (65.3%) [18], Bushullo village, southern Ethiopia (67.3%) [19], and southwest Nigeria (83.3%) [20]. This might be due to the fact that in southern Ethiopia, there is high humidity and rain throughout the year. Southern Nigeria is also located in the rain forest zone, possibly with high rain and humidity.

In the current study, the prevalence of hookworm species was 33.2% (95% CI: 28.9%–37.8%). This is consistent with findings in southwest Ethiopia (30.0%) [19] and Butajira, Ethiopia (36.1%) [21]. However, the current prevalence of hookworm species was higher than reports from Nigeria (16.8%) [22], Peninsular Malaysia (7.4%) [21], Brazil (12.6%) [15], and Thailand (6.6%) [16]. This variation might be attributed to differences in socioeconomic status and WASH practice of the people.

The current prevalence of *A. lumbricoides* was 4.3% (95% CI: 2.7%–6.6%). It was lower than an earlier studies in Mecha district (8.5%) [23], Bushullo village (37.2%) [19], Benjemaji zone (16.4%) [18], and southern Nigeria (67.7%) [20]. This difference might be due to the differences in altitude and location of the current study area, which is relatively hot and dry. In the current study, *T. trichuria* was not identified in the area. This might be because *T. trichiura* eggs are less resistant to temperature than those of *A. lumbricoides*, and this study, also conducted in dry environmental conditions during the study period (March-May), might have contributed to the absence of *T. trichiura* since the ova of this parasite is shown to be liable to destruction due to its fragility. Another reason could be that the egg is exposed to harsh environmental factors such as high soil temperature and lack of humidity during its development in the soil, which can impede development and cause the eggs not to develop to the infective stage [24].

The current study also showed that the majority of STH infections were under the category of light, with the exception of a few-moderate intensity of hookworm species infections. It is consistent with a study done in a rural community in southwest Ethiopia in which all hookworm species infections were light intensity [18]. This low intensity infection might be related to periodic deworming programs that could ultimately reduce worm burdens among the infected individuals.

In the present study, STHs infection among illiterate household members was higher (39.7%) than among those who had a secondary and above educational level. This finding is supported by previous research in northeastern Brazil, which found that STHs infection increased with decreasing education level [15]. This might be due to a low level of knowledge or awareness about the transmission of STHs among illiterates. The prevalence of STHs was also higher in farmers (37.4%) than in government employees and students. This is supported by a previous study in Nigeria [13]. This might be because those farmers have more soil contact for agricultural activity, which might increase the risk of STHs infection.

In the current study of STHs infection, study participants with families larger than five had significantly higher rates of infection. Study participants with family members greater than five were 1.74 times more likely to be infected by STHs than who had family members less than or equal to five (AOR = 1.74; 95% CI: 1.15–2.60; *P* = 0.008). It is supported by a result in southwest Ethiopia [18]. Because the study participants were household contacts of STH-infected patients in our study, they might share similar sources. Moreover, several STH species show household and genetic clustering patterns of transmission.

Environmental sanitation and personal hygiene are two of the most important components for STH control and elimination. The prevalence of STH in the study area was significantly higher among household members who had no latrine (open field defecation) compared to those who had a latrine (48% vs. 32%, respectively). Thus, household members who had no latrine around their houses had a 1.86 times higher odds of STH infection than those who had a latrine around their house (AOR = 1.86; 95% CI: 1.170%–2.90%; *P* = 0.02). This finding is supported by studies conducted in southwest Ethiopia (73.3% and 54.8%) [18], Nigeria (60.0% and 39.0%) [13], and northeastern Brazil (25.1% and 21%) [15]. If there is no latrine, the household members defecate in open fields and hence contaminate the environment, which increases the risk of STH infection.

## 5. Conclusion

The prevalence of soil transmitted helminths infections was high in Burie Zuria district, with hookworm species predominating. The absence of latrine and larger family sizes were significant predictors of STHs infection. Almost all (96.9%) hookworm species and all *A. lumbricoides* infections were categorized under light intensity of infection. Based on the findings, we recommended that all-inclusive community-based health education and mass preventive chemotherapy for the community should be implemented. Furthermore, interventions should be applied to improving the safety of water, accessibility, and utilization of latrine to strengthen STH prevention and control measures.

## Figures and Tables

**Table 1:** Sociodemographic characteristics of household contacts (*n* = 422) in Burie Zuria district, northwest Ethiopia, from March to May 2021.

Variables	Category		Frequency	%
Sex	Male		193	45.7
	Female		229	54.3
Age in years	<5		35	8.3
	5-15		129	30.6
	>15		258	61.1
Residence	Rural		413	97.9
	Urban		9	2.1
Family size	≤5		263	62.3
	>5		159	37.7
Educational status	Illiterate		199	47.2
	Informal education		26	6.2
	Primary school		162	38.4
	Secondary school		31	7.3
	College and above		4	0.9
Occupation	Farmer		227	53.8
	Student		158	37.4
	Merchant		7	1.7
	Government employee		3	0.7
	Others		27	6.4
Address (Kebele)	Alefa catchments	Alefa Basi	103	24.4
		Zalema	65	15.4
		Wadera Gendeba	36	8.5
		Adelagata	21	5
		Subtotal	225	53.3
	Tiatia catchment	Tiyatiya	94	22.3
		Shaqua Kebesa	64	15.2
		Denbun	39	9.2
		Subtotal	197	46.7

**Table 2:** Prevalence of intestinal parasites among household contacts (*n* = 422) in Burie Zuria district, northwest Ethiopia, from March to May 2021.

Parasites species		Frequency	%	95% CI
Helminths	Hookworm species	140	33.2	28.9-37.8
	*A. lumbricoides*	18	4.3	2.7-6.6
	Taenia species	4	0.9	0.4-2.4
	*H. nana*	2	0.5	0.1-1.7
Protozoal	*G. lamblia*	24	5.7	3.9-8.3
	*E. histolytica/dispar*	19	4.5	2.9-6.9
Total		199	47.2	42.4-51.9

**Table 3:** Prevalence of STH infections among household contacts (*n* = 422) in Burie Zuria district, northwest Ethiopia, from March to May 2021.

Catchment	Subkebeles	No_ of examined	Hookworm species *n* (%)	*A. lumbricoides* *n* (%)	Total *n* (%)
Alefa catchment	Alefa Basi	103	41 (39.8)	6 (5.8)	45 (43.7)
	Zalema	65	17 (26.2)	3 (4.6)	19 (29.2)
	Wadera	36	14 (38.9)	1 (2.8)	15 (41.7)
	Adelagata	21	7 (33.3)	0 (0)	7 (33.3)
	Subtotal	225	79 (35.1)	10 (4.4)	86 (38.2)
Tiatia catchment	Tiatia	94	35 (37.2)	5 (5.3)	39 (41.5)
	Shaqua K	64	16 (25.0)	3 (4.7)	19 (29.7)
	Denbun	39	10 (25.6)	0 (0)	10 (25.6)
	Subtotal	197	61 (31.0)	8 (4.1)	68 (34.5)
	**Total**	**422**	**140 (33.2)**	**18 (4.3)**	**154 (36.5)**

**Table 4:** Distribution of STH across various factors among household contacts (*n* = 422) in Burie Zuria district, northwest Ethiopia, from March to May 2021.

Variables	Category	Total examined	STHs negative *n* (%s)	STHs positive *n* (%)
Sex	Male	193	129 (66.8)	64 (33.2)
	Female	229	139 (60.7)	90 (39.3)
Age in years	<5	35	22 (62.9)	13 (37.1)
	5-15	129	86 (66.7)	43 (33.3)
	<15	258	160 (62.4)	98 (38.0)
Residence	Rural	413	262 (64.2)	151 (36.7)
	Urban	9	6 (66.7)	3 (33.3)
Family size	≤5	263	81 (68.8)	82 (31.2)
	>5	159	87 (54.7)	72 (45.3)
Educational status	Illiterate	199	120 (60.3)	79 (39.7)
	Informal education	26	17 (65.4)	9 (34.6)
	Primary school	162	106 (65.4)	56 (34.6)
	Secondary school	31	22 (71.0)	9 (29.0)
	College and above	4	3 (75.0)	1 (25.0)
Occupational status	Farmer	227	142 (62.6)	85 (37.4)
	Student	158	104 (65.9)	54 (34.1)
	Merchant	7	4 (57.2)	3 (42.8)
	Government employee	3	2 (66.7)	1 (33.3)
	Others	27	16 (59.3)	11 (40.7)
Latrine availability	Yes	326	218 (67.9)	108 (32.1)
	No	96	50 (52.1)	46 (47.9)
Open defecation	No	284	188 (66.0)	96 (34.0)
	Sometimes	25	20 (80.0)	5 (20.0)
	Yes	113	61 (54.0)	52 (46.0)
Washing of vegetables	Yes	378	243 (64.3)	135 (35.7)
	No	44	25 (56.7)	19 (43.3)
Source of drinking water	Tap water	9	6 (66.7)	3 (33.3)
	Well water	285	180 (63.2)	105 (36.8
	Stream/river	128	80 (62.5)	48 (37.5)
Handwashing before meal	Always	394	255 (64.7)	139 (35.3)
	Sometimes	21	11 (52.4)	10 (47.6)
	Never	7	3 (42.9)	4 (57.1)
Handwashing after toilet	Always	113	66 (58.4)	47 (41.6)
	Sometimes	72	43 (59.7)	29 (40.3)
	Never	217	140 (64.5)	77 (35.5)
Shoe-wearing habit	Always	249	162 (65.1)	87 (34.9)
	Sometimes	126	78 (61.9)	48 (38.1)
	Never	47	29 (61.7)	18 (38.3)

**Table 5:** Univariate and multivariate analysis of factors associated with STH among household contacts (*n* = 422) in Burie Zuria district, northwest Ethiopia, from March to May 2021.

Variables	Category	Total	Positive for STH	COR(95% CI)	*P* value	AOR(95% CI)	*P* value
Sex	Male	193	64 (33.2)	1			
	Female	229	90 (39.3)	1.20 (0.81-1.79)	0.369		
Age in years	<5	35	13 (37.1)	1			
	5-15	129	43 (33.3)	0.85 (0.39-1.84)	0.674		
	>15	258	98 (38.0)	1.04 (0.50-2.15)	0.923		
Residence	Urban	9	3 (33.3)	1			
	Rural	413	151 (35.8)	1.15 (0.28-4.68)	0.842		
Family size	≤5	283	82 (31.2)	1			
	>5	159	72 (45.3)	1.83 (1.22-2.74)	**0.004**	1.74 (1.15-2.60)	**0.008**
Educational status	Illiterate	199	78 (39.2)	1.93 (0.20-18.93)	0.571		
	Informal education	26	9 (34.5)	1.59 (0.14-17.56)	0.706		
	Primary school	162	57 (35.2)	1.59 (0.16-15.59)	0.693		
	Secondary school	31	9 (29.0)	1.43 (0.13-15.52)	0.769		
	College & above	4	1 (25.0)	1			
Occupational status	Government employee	1	0	1			
	Student	158	54 (34.2	1.03 (0.09-11.71)	0.98		
	Merchant	7	2 (28.6)	0.80 (0.04-14.64)	0.88		
	Farmer	227	87 (38.9)	1.38 (0.11-17.09)	0.80		
	Others	27	11 (40.7)	1.22 (0.11-13.65)	0.87		
Presence of latrine Facility	Yes	326	107 (32.8)	1			
	No	96	46 (47.9)	1.74 (1.09-2.78)	**0.009**	1.86 (1.17-2.90)	**0.02**
Open defecation practice	No	284	96 (34.0)	1			
	Sometime	25	5 (20.0)	0.62 (0.59-2.41)	0.322		
	Yes	113	52 (46)	1.67 (1.17-2.67)	**0.024**	1.19 (0.59-2.40)	0.063
Wash vegetables before eating	Yes	378	136 (36)	1			
	No	44	18 (40.9	1.35 (0.71-2.55)	0.35		
Source of drinking water	Tap water	9	3 (33.3)	1			
	Well water	285	103 (36.1)	1.13 (0.28-4.62)	0.863		
	Stream	128	48 (37.5)	1.2 (0.29-5.02)	0.803		
Handwash before meal	Always	394	140 (35.5)	1			
	Sometime	21	10 (47.6)	0.41 (0.09-1.87)	0.252		
	Never	7	4 (57.1)	1.26 (0.12-3.83)	0.663		
Handwash after toilet	Always	133	48 (30.1)	1			
	Sometime	72	29 (40.3)	1.19 (0.66-2.15)	0.555		
	Never	217	77 (35.5)	0.94 (0.62-1.53)	0.909		
Shoe-wearing habit	Always	249	89 (35.7)	1			
	Sometime	126	46 (36.5)	1.03 (0.66-1.61)	0.884		
	Never	47	19 (40.4)	1.22 (0.65-2.31)	0.541		

**Table 6:** Frequency and intensity of STH infection in KK technique among household contacts (*n* = 422) in Burie Zuria district, northwest Ethiopia, from March to May 2021.

STHs	No. of positive in KK	Mean egg count per gram	Intensity of the STHs infections		
			Light *n* (%)	Moderate *n* (%)	Heavy *n* (%)
Hookworm species	129	927	125 (96.9)	4 (3.1)	0
*A. lumbricoides*	18	408	18 (100)	0	0

## Data Availability

The datasets analyzed during the current study are not publicly available due to institutional regulation but are available from the corresponding author on reasonable request.

## Abbreviations

AOR: Adjusted odds ratio
COR: Crude odds ratio
DALYs: Disability-adjusted life years
EPG: Eggs per gram
FECT: Formol-ether concentration technique
KK: Kato-Katz
MDA: Mass drug administration
SPSS: Stastical package for social sciences
STHs: Soil-transmitted helminths
WASH: Water access, sanitation and hygiene
WHO: World Health Organization
